# *Trans*-gastric and *trans*-abdominal percutaneous drainage of acute peripancreatic fluid infected collections: A retrospective analysis

**DOI:** 10.1016/j.amsu.2022.104080

**Published:** 2022-06-25

**Authors:** Carlos Rey, Danny Conde, Felipe Girón, Daniela Ayala, Juliana Gonzalez, Daniela Melo, Marco Quintero

**Affiliations:** a Hospital Universitario Mayor Méderi, Colombia; b Universidad el Rosario, Colombia

**Keywords:** Acute pancreatitis, Radiology interventional, Drainage, Peripancreatic necrosis

## Abstract

**Background:**

Acute pancreatitis is one of the most common gastrointestinal diseases. Approximately 20% of the patients develop peripancreatic collections. Step-up management it's now the best approach with less rate of morbidity and mortality compared with open or minimally invasive surgery. Percutaneous management could reach a success rate between 50 and 76%. Our study shows the outcomes of *trans*-gastric versus transabdominal percutaneous drainage in cases of acute peripancreatic fluid infected collections in the absence of interventionist endoscopy.

**Methods:**

A retrospective review of a prospectively collected database was conducted. All the patients older than 18 years old that underwent percutaneous drainage between January 2010–December 2021 were included. Analysis and description of outcomes such as mortality, complications, and avoidance of surgical procedures was performed.

**Results:**

18 patients underwent percutaneous drainage. 66.67% of patients were male. Mean age was 52.55 ± 22.06 years. Mean weight was 74.43 ± 15.25 kg. Mean size of peripancreatic collections 118.4 ± 49.12 mm. Wall-off necrosis was present in 33.33%. *Trans*-gastric approach was performed in 50% of the cases, the rest was *trans*-abdominal. No mortality was evidenced after 30 days of follow up. After *trans*-gastric percutaneous drainage, all patients avoided surgical open or laparoscopic procedure.

**Conclusion:**

Standardized step-up approach shows increased rates of success in percutaneous drainage of peripancreatic collections. Our case series shows a high rate of success in terms of avoidance any surgical procedure with no mortality after *trans*-abdominal and *trans*-gastric percutaneous drainage. Nevertheless, further prospective studies with higher sample size are needed.

## Highlights

• *Trans*-gastric and *trans*-abdominal percutaneous drainage of peripancreatic infected collections are safe and feasible procedures.
• In cases of no access to interventional endoscopy, percutaneous drainage remains to be a safe procedure.
• Multidisciplinary approach leads to good outcomes with less rate of morbidity and mortality.

## 1 Background

Acute pancreatitis (AP) is defined as an inflammatory process of the pancreatic gland associated to local and systemic inflammatory response, being one of the most common gastrointestinal diseases with an incidence of 34 per 10.0000 habitants per year in high income countries [1]. In our country, there is no specific epidemiological data, therefore it has not been possible to establish the real incidence of the disease and its management outcomes [2].

Around 80% of the acute pancreatitis are attributed to gallstones, followed by alcohol in 9% of the cases and 5% secondary to traumatic events; the remaining percentage is due to other multiple etiologies including pharmacological induced among others [3]. Acute pancreatitis severity is highly variable, ranging from self-limited episodes to multiorgan failure [4,5]. Almost 20% of the patients with acute pancreatitis develop a complicated clinical course that can progress and lead to fatal outcomes [6,7]. Despite the etiological differences, mortality rates are about 4–5% in mild acute pancreatitis and 30–50% in severe pancreatitis [8].

The imaging evaluation depends on the medical criteria and the clinical evolution of the patient. These are usually suggested when there is diagnostic doubt, deterioration, multiple organ failure and suspected complications [[9], [10], [11]]. The abdominal ultrasound, computerized axial tomography (CT) and the magnetic resonance (MR) are essential for determining the etiology, however, ultrasound lacks efficacy due to the frequent interposition of gas; while the TC is the gold standard for diagnosis, classification, and identification of complications [9]. Imaging findings are interpreted in the context of severity using the tomographic severity index (CTSI).

Treatment of acute pancreatitis has evolved over the years, currently it is based on has 4 pillars: early intravenous hydration, adequate nutrition, analgesia, and additional necessary interventions. Although consensus has been reached on the nomenclature, there are still no definitive agreements on treatment options [8,9,12]. Once a torpid clinical progression of patients, complications must be ruled out differentiating them into systemic (transitory and persistent organ failure) and local complications. The Atlanta consensus (1992), generated a universally nomenclature that classified local complications into 5 types, based on imaging findings: Peripancreatic acute liquid collections, acute necrotic collection, pancreatic pseudocyst, walled-off necrosis, and infected necrosis [[8], [9], [10], [11], [12]].

Adequate drainage of infected necrotic collections is the fundamental pillar in the treatment of these patients, in conjunction with opportune antibiotic therapy and multidisciplinary medical management [[8], [9], [10], [11], [12]]. Percutaneous drainage is routinely performed guided by computed tomography and has represented an advance in the management of peripancreatic collections [[13], [14], [15]]. Its use is recommended in the literature mainly as a bridging therapy in those patients with infected necrosis in whom it is necessary to stabilize the septic process before considering surgical management, although recent studies have shown efficacy of approximately 40% as the only treatment [[13], [14], [15]]. The percutaneous management of the collections control the origin of the infection by removing the infected fluid, reducing surgical stress and the complications associated with conventional interventions [15]. The success rate of percutaneous drains varies between 14 and 86% with low mortality and morbidity, and accompanied by drains rigorous care, correct cleaning and frequent irrigation could improve even more the result of the technique [[14], [15], [16], [17]]. The approach to pancreatic collections is initially considered as a minimally invasive procedure since lower rates of complications have been demonstrated. Percutaneous procedures can be performed in the earliest stages of the evolution, with relative safety and effectiveness, reducing local complications and the patient's systemic inflammatory response [13,14,16]. The aim of this study, it's to describe the experience and outcomes in management of infected pancreatic necrosis by percutaneous drainage (*trans*-abdominal and *trans*-gastric approach) in an institution in which interventionist endoscopy it's not available.

## 2 Materials and methods

### 2.1 Study population

**Fig. 1:**
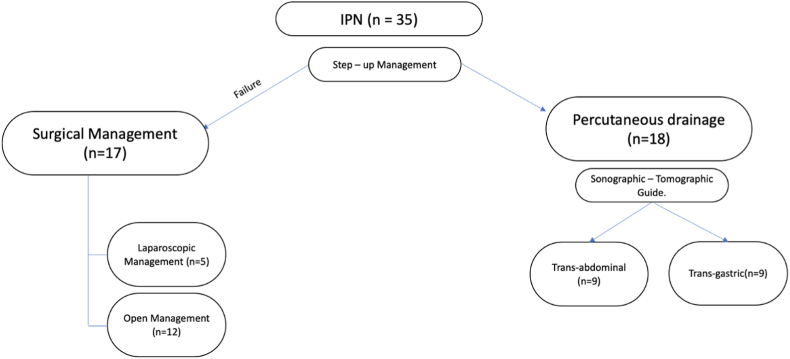
Flow - chart decision process IPN: *Infected pancreatic necrosis.*

With the Institutional Review Board's approval and following Health Insurance Portability and Accountability Act (HIPAA) guidelines, a retrospective review of a prospectively collected database was conducted. The present study has been reported in line with PROCESS guidelines [17] All patients over 18 years of age that underwent percutaneous drainage of peripancreatic infected collections due to acute biliary pancreatitis between January 2014 and May 2021 were included (Fig. 1). Patients with no post-drainage evolution and missing data were excluded. Ethical compliance with the Helsinki Declaration, current legislation on research Res. 008430-1993 and Res. 2378-2008 (Colombia) and the International Committee of Medical Journal Editors (ICMJE) were ensured under our Ethics and Research Institutional Committee (IRB) approval.

### 2.2 Data management

Descriptive statistics were reported in terms of variable nature. Qualitative analysis was performed in terms of frequencies and percentages, while quantitative analysis was done in terms of mean and standard deviations of normally distributed data and medians and interquartile ranges (IQRs) for non-normally distributed data. Statistical analysis were performed to describe the association between the approach and the main outcomes.

### 2.3 Percutaneous management

#### 2.3.1 Drainage indication

In patients presented with suspected acute local infected complications due to acute biliary pancreatitis such as peripancreatic collections, or necrotic infected collections were assessed by abdominal computed tomography. Once confirmed the presence of an infected collection (defined by clinical deterioration, increased inflammatory signs, and elevation of laboratory markers such as white blood cells, reactive C protein, elevated procalcitonin and presence of gas in the peripancreatic collection) an evaluation by single interventionist radiologist was requested to perform percutaneous drainage.

#### 2.3.2 Technique

**Image 1:**
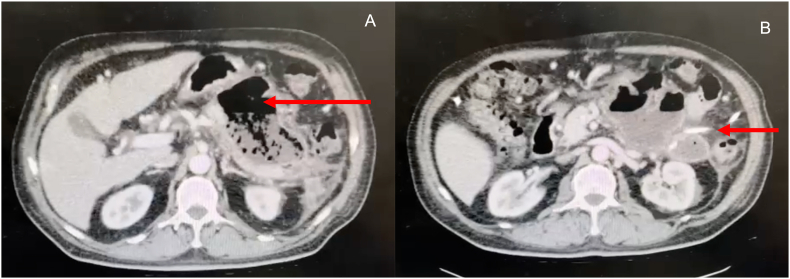
A.Peripancreatic infected collection with gas and perpipancreatic edema.(Red arrow) B Peripancreatic infected collection with percutaneous drainage (Red arrow).

Drainage was performed with imagenologic guidance (all procedures were performed with a sonographic guide initially, and in technically difficult cases, with a tomographic guide.). In sterile conditions, local/general anesthesia was required according to clinical status of the patient. Seldinger technique was used by protocol; a 0.8-mm hydrophilic J-shaped guidewire were used to perform the adequate placement of the catheter into the infected collections. *Trans*-abdominal or *trans*-gastric management were performed, depending on the localization and technical availability to perform the procedure. In cases of *trans*-gastric drainage, a 16–18 French pig-tail catheter drainage was preferred, and in *trans*-abdominal cases, a 12–16 French pigtail was used. After the placement, aspiration and culture of the collection was performed; and later, irrigated with 1–3 L of saline solution. Requirement of new drainage was defined by non-improvement in clinical condition, and tomographic evidence of collection persistence (Image 1).

#### 2.3.3 Follow up

Patients were assessed until 1 h after the procedure, to avoid immediate post-procedure complications. All patients were evaluated by a general surgeon after the procedure, and with close evaluation of vital signs, arterial gasometry, and clinical assessment. In cases of Intensive care unit stay, a closer surveillance was performed. Post - procedure tomography only was performed if patients have a worse clinical course. All patients were evaluated after resolution of the complication in external valoration by a general surgeon.

## 3 Results

### 3.1 Demographic characteristics

Between 2014 and 2021, 1020 episodes of acute biliary pancreatitis, and 35 patients present infected peripancreatic collections with or without necrosis. 18 patients underwent percutaneous drainage and were included in the study, the remaining patients (n = 17) technical difficulties don't allow the percutaneous intervention and require surgical management (open and laparoscopic debridation). 66.67% (n = 12) of patients were male. Mean age was 52.55 ± 22.06 years. Mean weight was 74.43 ± 15.25 kg. History of arterial hypertension was presented in 22.22% (n = 4), type 2 diabetes mellitus in 5.5%.(n = 1) (See Table 1). In all cases etiology of pancreatitis was biliary.

**Table 1:** Demographic characteristics.

Variable	Result	
Gender % (n)		
**Male**	66.6(12)	
**Female**	33.3(6)	
	**Mean**	**SD**
**Age**	52.5	22.06
**Weight**	74.4	15.25
**Comorbidities %(n)**		
**No-Comorbidities**	66.6(12)	
**Arterial Hypertension**	22.2 (4)	
**Type 2 Diabetes**	5.5(1)	
**Chronic Renal disease**	5.5(1)	

### 3.2 Peripancreatic collection characteristics

All patients were presented with acute complications according to Atlanta classification (<4 weeks) and were classified as acute peripancreatic fluid collection (APFC). Computed tomography severity index (CTSI) score was assessed, and classified as: mild (0–3), moderate (4–6), and severe (7-10). Majority of the patients 83.33% (n = 14) presented with a severe CTSI score. Fluid characteristics based on tomography images were defined as: heterogeneous or homogeneous; in our population, in most of the cases, collection was heterogeneous. (83.33%). As well, pancreatic necrosis was evaluated and classified > or equal than 30%; in the patients included in the study, most of the patients present with more than 30% of pancreatic necrosis (61.11%; n = 10). Size of the peripancreatic collection was calculated based on tomographic findings with a mean size of 118.4 ± 49.12 mm. Wall-off necrosis was present in 33.33% (n = 6). In all cases, fluid collections were cultivated; findings are summarized in Table 2.

**Table 2:** Peripancreatic collection characteristics.

Variable	Result	
CTSI % (n)		
Mild	16.6(3)	
Moderate	0 (0)	
Severe	83.3 (15)	
**Fluid % (n)**		
Heterogeneous	83.3 (15)	
Homogeneous	16.6(3)	
**Pancreatic necrosis % (n)**		
<30%	38.8 (7)	
>30%	61.1 (11)	
**Type of collection % (n)**		
Peripancreatic Collection	66.6 (12)	
Wall-off Necrosis	33.3 (6)	
**Culture % (n)**		
E. Coli	50 (9)	
K. Pneumoniae	33.3 (6)	
Lactococcus	5.5 (1)	
Candida Albicans	11.1 (2)	
	**Mean**	**SD**
**Size of the collection**	118.27 mm	49.12

### 3.3 Outcomes

Multi-organic dysfunction was present in 94.4% (n = 17) of the patients prior to percutaneous drainage. After the procedure, only 5.5% (n = 1) of the cases did not modify clinical conditions of organic dysfunction. In 94.4% (n = 17) of the population, percutaneous drainage was considered successful, by avoiding pancreatic surgery (debridation, necrosectomy, laparoscopic debridation). Number of required drainages were evaluated as well, the majority of the patients (87.50%; n = 15) only needed one procedure, 6.25% (n = 2) required two, and 6.25% (n = 2) required three percutaneous drainages. In all cases, only one pigtail catheter was placed in the collection. Tomographic guide was the most frequently used, in 83.33% (n = 14) of the cases. *Trans*-gastric approach was performed in 50% (n = 9) of the cases, the rest was *trans*-abdominal. No mortality was evidenced after 30 days of follow up. Pancreatic fistula was observed in 6,25% (n = 2) of the cases; no other complications were observed. Management of drains were teached to the patients, and all follow up was performed by a pancreatic surgeon out-hospital.

### 3.4 Trans - abdominal versus *trans*-gastric drainage

After *trans*-gastric percutaneous drainage, all patients avoided surgical open or laparoscopic procedure; compared with *trans*-abdominal approach in that one patient's procedure was considered unsuccessful, and patient required laparoscopic drainage of infected pancreatic necrosis. There was a slight difference in the approach in terms of improvement of organ dysfunction. (*Trans*-abdominal 1 patient improves organ dysfunction compared with 0 in *trans*-gastric approach). Sonographic approaches were preferred in cases of *trans*-abdominal drainage, and tomographic approaches were the most frequent guide in cases of *trans*-gastric procedure. (See Table 3). Statistical analyses were performed in terms of avoidance of surgery and complications, however, there is no statistical difference of the approaches. (p = 0.12, p = 0.4, CI 95% Respectively).

**Table 3:** *Trans*-abdominal versus *Trans*-gastric percutaneous drainage.

Variable	TAPD	TGPD
**Successful drainage % (n)**	88.8 (8)	100 (9)
**Organ dysfunction improvement % (n)**	12.5 (1)	0 (0)
**Required surgical procedure % (n)**	12.5 (1)	0 (0)
**Tomographic approach % (n)**	77.8 (7)	88.8 (8)
**Sonographic approach % (n)**	25% (2)	12.5 (1)
**Required drainages Mean (sd)**	1.44 (0.72)	1(0)

## 4 Discussion

Evolution of AP into necrotizing pancreatitis has an increased rate of mortality, reaching 35% in some cases of series [18,19]. Effectiveness in the treatment of peripancreatic collections associated with necrotic tissue are well described in the literature; in previous years, open necrosectomy was considered the gold standard in the management of this complication [19], however, minimally invasive approach was explored to avoid surgery, and prevent surgical morbidity [19]. PANTER trial [19,20], demonstrate the numerous benefits of an “step - up” approach over open surgery; with lesser proportion of complications, reduced time of recovery, and less rate of mortality in cases of percutaneous drainage [19,20]; also, PENGUIN trial, shows benefit of endoscopic approach over surgery in the same postoperative outcomes [21]. Surgical approach shows an increased rate of mortality, reaching almost 42% in some series reported [19]. For that reason, the step-up approach in general surgery departments should be standardized to reduce mortality rates and prevent chronic morbidity.

Success rate of percutaneous drainage is in a range of 0–78% in some series of cases [22], and it is referred to most of the times in terms of avoiding surgical procedures or mortality rates [22]. Freeny et al. [23], was the first author to describe the effectiveness of percutaneous drainage and shows a success rate of 47% in well selected cases and be able to decrease the multi-organ failure [23]. However, success rate is related to a multidisciplinary approach, gathering interventionist radiologists, gastroenterologists, general surgeons, pancreatic surgeons, and intensive care units’ members [23]. Sharma et al. [22], report a high rate of success in CT guided percutaneous drainage, with 85,7%, avoiding surgical approach in 75% of the cases. As well, Wronski et al. [24], shows a 33% of success rate in ultrasound guided percutaneous drainage, and could be used as a definitive approach to avoid surgery and reach complete resolution of the complication [24]. These results are comparable with ours, with a success rate of 94.4% of the cases, with no complications after 30 days of follow up.

Tomographic guide it's the most frequent one used; only 4 authors described their experience using ultrasound guide in peripancreatic drainage [24]; in our population no differences between the radiologic approach, however, it's important to declare that in cases of increased technical difficulty CT guide was preferred over ultrasound, depending on the radiologist experience.

Is well known that infected peripancreatic collections are related with bacterial infection [22], in the majority of the cases, E.Coli and and E. Faecioum were the most frequent bacteria isolated [22]. In our population, no gram-positive bacteria were obtained, and E. Coli remains to be the most persistent bacteria isolated; however, fungi are a microbiological concern that needs to be in our minds in critically ill patients as we can see in our population with 11.1% of the cases.

The use of more than one catheter, it's related to increased success rates in patients with large collections with infected necrosis, lavage of the cavity could be related with better outcomes because, the use of saline solution could reduce the rate of debris and necrotic tissue that is susceptible of infection [[22], [23], [24]].

Some literature reports the trans gastric percutaneous approach in the drainage of pancreatic pseudocyst and collections [[25], [26], [27]], however, with the advent of endoscopic procedures, these interventions are abandoned, and replaced with endoscopic ultrasound guided drainage or cystogastrostomy [[25], [26], [27]]. Nevertheless, in some low-income countries, interventionist endoscopy is not available, and expertise in interventionist radiologists have an increased importance in the management of these conditions.

In our population, *trans*-gastric approach shows comparable results with *trans*-abdominal approach, with high rate of success, and achieves a 100% rate of avoiding surgical procedures, with no complications after 30 days of follow up. To the best of our knowledge, our study is the first one to describe the trans gastric approach in the management of percutaneous infected necrosis, with an increased rate of success, comparable with endoscopic approach according to the present-day literature. In low-income countries, access to interventionist endoscopy could be limited, and the radiologist techniques could be a feasible approach in acute complications of pancreatitis.

Limitations of our study, includes the retrospective nature, and the limited number of cases (related with the proportion of patients that develop infected pancreatic necrosis), as well, in our center the non-availability of ultrasound-endoscopic guide to perform endoscopic procedures could limit the comparison of the approach.

## 5 Conclusion

Standardized step-up approach shows increased rates of success in percutaneous drainage of peripancreatic collections. Our case series shows a high rate of success in terms of avoidance any surgical procedure with no mortality, and any differences were observed in clinical outcomes between *trans*-gastric or *trans*-abdominal approach. Nevertheless, further prospective studies with higher sample size are needed.

## Ethical approval

Ethical approval of Hospital Universitario Mayor Méderi and Universidad el Rosario was reached.

## Sources of funding

Authors do not have any founding.

## Author contribution

CR, DC, MQ Conception of the idea, manuscript edition and final approval, DA, JG Data analysis, manuscript writing.

## Registration of research studies

Name of the registry:

Unique Identifying number or registration ID:

Hyperlink to your specific registration (must be publicly accessible and will be checked).

## Guarantor

Carlos Rey.

## Consent

Informed consent of each patient was obtained.

## Declaration of competing interest

Authors do not have any conflict of interest.

## References

[1] Boxhoorn L., Voermans R.P., Bouwense S.A., Bruno M.J., Verdonk R.C., Boermeester M.A., van Santvoort H.C., Besselink M.G. (2020). Acute pancreatitis. Lancet.

[2] Riveros R., Nieto J.A., Vargas F. (2008). https://repository.urosario.edu.co/bitstream/handle/10336/1019/Pancreatitis%20aguda.pdf.

[3] Forsmark C.E., Baillie J., Practice A.G.A.I.C., Economics C., Board A.G.A.I.G. (2007). AGA Institute technical review on acute pancreatitis. Gastroenterology.

[4] Colvin S.D., Smith E.N., Morgan D.E., Porter K.K. (2020). Acute pancreatitis: an update on the revised Atlanta classification. Abdom Radiol (NY).

[5] Nieto J.A., Rodríguez S.J. (2019). Manejo de La pancreatitis Aguda: Guía de práctica clínica Basada En La Mejor información Disponible. Rev Colomb Cir.

[6] Pérez F., Arauz Valdes E. (2020). Pancreatitis Aguda: Artículo de Revisión. Rev méd cien t..

[7] Colvin S.D., Smith E.N., Morgan D.E., Porter K.K. (2020). Acute pancreatitis: an update on the revised Atlanta classification. Abdom Radiol (NY).

[8] Jinno N., Hori Y., Naitoh I., Miyabe K., Yoshida M., Natsume M., Kato A., Asano G., Sano H., Hayashi K. (2019). Predictive factors for the mortality of acute pancreatitis on admission. PLoS One.

[9] Lankisch P.G., Apte M., Banks P.A. (2015). Acute pancreatitis. Lancet.

[10] Rodríguez E.B.M. (2018). https://repositorio.unal.edu.co/bitstream/handle/unal/62919/Caracterización%20de%20los%20pacientes%20con%20Pancreatitis%20Aguda%20que%20ingresan%20al%20HUN%20Final.pdf?sequence=1&isAllowed=y.

[11] Paulino J., Ramos G., Veloso Gomes F. (2019). Together we stand, Divided we fall: a multidisciplinary approach in complicated acute pancreatitis. J. Clin. Med..

[12] Mancilla C.A., Sanhueza A.S. (2010). Clasificación de Balthazar-Ranson. Gastroenterol. Latinoam.

[13] Pérez Chaca G. (2017). http://revistaintervencionismo.com/wp-content/uploads/INT_2016_048_Revision.pdf.

[14] an Santvoort H.C., Bakker O.J., Bollen T.L., Besselink M.G., Ali U.A., Schrijver A.M. (2011). A conservative and minimally invasive approach to necrotizing pancreatitis improves outcomes. Gastroenterology.

[15] Bennett S., Lorenz J.M. (2012). The role of imaging-guided percutaneous procedures in the multidisciplinary approach to treatment of pancreatic fluid collections. Semin. Intervent. Radiol..

[16] Hollemans R.A., Bollen T.L., van Brunschot S., Bakker O.J., Ali U.A., van Goor H. (2016). Predicting success of catheter drainage in infected necrotizing pancreatitis. Ann. Surg..

[17] Agha R.A., Sohrabi C., Mathew G., Franchi T., Kerwan A., O'Neill N., PROCESS Group (2020). The PROCESS 2020 guideline: updating consensus preferred reporting of CasESeries in surgery (PROCESS) guidelines. Int. J. Surg..

[18] Fabbri C., Luigiano C., Maimone A., Polifemo A.M., Taran- tino I., Cennamo V. (2012). Endoscopic ultrasound-guided drainage of pancreatic fluid collections. World J. Gastrointest. Endosc..

[19] Varadarajulu S., Lopes T.L., Wilcox C.M., Drelichman E.R., Kilgore M.L., Christein J.D. (2008). EUS versus surgical cyst-gastros- tomy for management of pancreatic pseudocysts. Gastrointest. Endosc..

[20] Sion M.K., Davis K.A. (2019). Step-up approach for the management of pancreatic necrosis: a review of the literature. Trauma Surg. Acute Care Open.

[21] Besselink M.G., van Santvoort H.C., Nieuwenhuijs V.B., Boermeester M.A., Bollen T.L., Buskens E., Dejong C.H., van Eijck C.H., van Goor H., Hofker S.S., Lameris J.S., van Leeuwen M.S., Ploeg R.J., van Ramshorst B., Schaapherder A.F., Cuesta M.A., Consten E.C., Gouma D.J., van der Harst E., Hesselink E.J., Houdijk L.P., Karsten T.M., van Laarhoven C.J., Pierie J.P., Rosman C., Bilgen E.J., Timmer R., van der Tweel I., de Wit R.J., Witteman B.J., Gooszen H.G. (2006). Dutch Acute Pancreatitis Study Group. Minimally invasive 'step-up approach' versus maximal necrosectomy in patients with acute necrotising pancreatitis (PANTER trial): design and rationale of a randomised controlled multicenter trial [ISRCTN13975868]. BMC Surg..

[22] Bakker O.J., van Santvoort H.C., van Brunschot S. (2012). Endoscopic transgastric vs surgical necrosectomy for infected necrotizing pancreatitis: a randomized trial. JAMA.

[23] Sharma P., Sharma S., Yadav A., Rotem E. (2019). CT guided percutaneous drainage in necrotizing pancreatitis - highly successful in appropriately selected patients - single center experience. J. Pancreas.

[24] Freeny P.C., Hauptmann E., Althaus S.J., Traverso L.W., Sinanan M. (1998). Percutaneous CT-guided catheter drainage of infected acute necrotizing pancreatitis: techniques and results. AJR Am. J. Roentgenol..

[25] Wroński M., Cebulski W., Karkocha D., Słodkowski M., Wysocki L., Jankowski M., Krasnodębski I.W. (2013). Ultrasound-guided percutaneous drainage of infected pancreatic necrosis. Surg. Endosc..

[26] Davies R.P., Cox M.R., Wilson T.G. (1996). Percutaneous cystogastrostomy with a new catheter for drainage of pancreatic pseudocysts and fluid collections. Cardiovasc. Intervent. Radiol..

[27] Van Sonnenberg E., Wittich G.R., Casola G., Branningan T.C., Karnel F., Stabile B.E., Varney R.R., Christensen R.R. (1989). Percutaneous drainage of infected and reinfected pancreatic pseudocysts: experience in 101 cases. Radiology.

